# Editorial: Multi-targeted tyrosine kinase inhibitors in the treatment of cancer and neurodegenerative disorders

**DOI:** 10.3389/fchem.2024.1460347

**Published:** 2024-07-26

**Authors:** Belgin Sever, Luciano Saso, Rumiana Tzoneva, Valentina Onnis, Halilibrahim Ciftci

**Affiliations:** ^1^ Department of Pharmaceutical Chemistry, Faculty of Pharmacy, Anadolu University, Eskisehir, Türkiye; ^2^ Medicinal and Biological Chemistry Science Farm Joint Research Laboratory, Faculty of Life Sciences, Kumamoto University, Kumamoto, Japan; ^3^ Department of Physiology and Pharmacology “Vittorio Erspamer”, Sapienza University of Rome, Rome, Italy; ^4^ Institute of Biophysics and Biomedical Engineering, Bulgarian Academy of Sciences, Sofia, Bulgaria; ^5^ Department of Life and Environmental Sciences, University of Cagliari, Monserrato University Campus, Cagliari, Italy; ^6^ Department of Bioengineering Sciences, Izmir Katip Celebi University, Izmir, Türkiye; ^7^ Department of Drug Discovery, Science Farm Ltd., Kumamoto, Japan

**Keywords:** tyrosine kinase Inhibition, cancer, apoptosis, downstream signalling, Abl and Src tyrosine kinases, epidermal growth factor receptor (EGFR), HER-2 (ErbB2)

Tyrosine kinases, which catalyse the phosphorylation of tyrosine residues in target proteins using ATP, play a plethora of roles in the regulation of diverse cellular functions including growth, motility, differentiation, and metabolism. Since their activity is tightly regulated in normal cells, in cancer due to emerging mutations, overexpression and autocrine paracrine stimulation, they can acquire transforming functions (Riegel et al., 2022). The pathogenesis of neurodegenerative diseases is also related with protein kinases (Kawahata and Fukunaga, 2023).

Tyrosine kinases are mainly classified as receptor tyrosine kinases and non-receptor tyrosine kinases, including crucial members. Epidermal growth factor receptor (EGFR) belongs to the ERbB family of receptor tyrosine kinases along with three other closely related receptors, namely, HER-2, HER-3, and HER-4. EGFR and HER-2 lead to autophosphorylation of the intracellular domain through tyrosine kinase activity and subsequent stimulation of downstream cascade that may result in proliferation, suppression of apoptosis, metastasis and angiogenesis. On the other hand, c-Abl (Abl-1) is a non-receptor tyrosine kinase, which is also essential in the regulation of several anti-apoptotic and proliferative signal transduction pathways. They have mainly been identified as important targets for several types of cancer such as EGFR for non-small-cell lung cancer (NSCLC), glioma and colorectal cancer; HER-2 for breast and colorectal cancers and c-Abl for chronic myeloid leukemia (CML).

One of the major platforms that they have participated in is neurodegenerative disorders such as Parkinson’s disease, Alzheimer’s disease and amyotrophic lateral sclerosis (ALS). Aberrant activity of tyrosine kinases, in particular EGFR and c-Abl, have been reported to induce neuronal apoptosis and cell cycle arrest in response to a wide range of stimuli resulting in neurodegeneration and neuroinflammation.

The main goal of the Research Topic entitled “*Multi-targeted Tyrosine Kinase Inhibitors in the Treatment of Cancer and Neurodegenerative Disorders*” is to identify new and promising tyrosine kinase inhibitors to be effective in cancer and neurodegenerative disorders.

In this Research Topic, twelve high-quality papers have been published (three of them original research articles, two reviews, one systematic review, five case reports and one clinical trial), which focused mainly on cancer. In these studies, the authors presented their latest results across a wide spectrum of research dealing with usage of multi-target tyrosine kinase inhibitors in the microsatellite stability subtype of colorectal cancer (Liu et al.), discussing the effects of anlotinib combined with chemotherapy in patients with metastatic triple-negative breast cancer (Huang et al.), reviewing the treatment of advanced hepatocellular carcinoma with multikinase inhibitor axitinib (Jiang et al.), as well as discussing the discontinuation and clinical outcomes in patients with B-cell lymphoproliferative diseases treated with bruton tyrosine kinase inhibitors (BTKi) in China (Yan et al.). Intriguing are work of (Li et al.), who demonstrated safety and efficacy of transarterial chemoembolization combined with tyrosine kinase inhibitors and camrelizumab in the treatment of patients with advanced unresectable hepatocellular carcinoma and work of (Wang et al.), who showed effective combined treatment of sunitinib (tyrosine kinase inhibitor) with immune checkpoint inhibitors. The role of tyrosine kinase inhibitors was also highlighted in pulmonary carcinoma by work of (Yang et al.), who used aumolertinib for effective treatment for asymptomatic pulmonary giant cell carcinoma with EGFR L858R mutation and by work of (Li et al.), who demonstrated the response to fifth-line brigatinib plus entrectinib in an anaplastic lymphoma kinase (*ALK*)-rearranged lung adenocarcinoma with an acquired ETV6-NTRK3 fusion. The successful and safety response to tyrosine kinase inhibitors was found in treatment of metastatic renal cell carcinoma (Krawczyk et al.) and in combination of two inhibitors bevacizumab with erlotinib for a novel FH gene mutation hereditary leiomyoma and renal cell carcinoma (Bai et al.). In the work of (Liu et al.), the molecular and microenvironment changes upon midostaurin treatment in mast cell leukemia at single-cell level were revealed. The effectiveness of using tyrosine kinase inhibitors and their challenges in glioblastoma treatment was discussed in the work of (Rahban et al.).

Together the published papers in this collection highlight the important role which tyrosine kinases play in pleotropic number of cellular processes in physiological and pathological conditions. The papers reveal the specificity and the effectiveness of using different tyrosine kinases inhibitors as a successful treatment of different cancers. It is hoped that the reader will find useful and appreciate this Research Topic.
